# Total hip arthroplasty in patients with severe hip dysplasia and congenital pubic diastasis: report of two cases

**DOI:** 10.1186/s12891-021-04702-x

**Published:** 2021-09-23

**Authors:** Christian Goetze, Filippo Migliorini, Christian Dominik Peterlein

**Affiliations:** 1 Department of Orthopaedics, Auguste-Viktoria Clinic, Ruhr University Bochum, 32545 Bad Oeynhausen, Germany; 2 Department of Orthopaedics, Trauma, and Reconstructive Surgery, RWTH University Hospital, Pauwelsstr. 30, 52074 Aachen, Germany

**Keywords:** Congenital pubic diastasis, Dysplasia, Arthroplasty, Bladder exstrophy

## Abstract

**Background:**

Congenital bladder exstrophy is a rare malformation which is often associated with pubic diastasis and hip dysplasia. We reported the case two patients who underwent total hip arthroplasty (THA) due to advanced osteoarthritis combined with large congenital pubic diastasis (> 10 cm).

**Case presentation:**

The first patient, a 39 years old woman with a pubic diastase and severe hip dysplasia on both sides was treated with a primary two-staged bilateral THA. Both hips were treated with a cementless osteoconductive cup (TM, Zimmer-Biomet) and a cementless stem (Alloclassic SL, Zimmer-Biomet). A 10° elevated rim liner of the cup was used in order to avoid dislocation. The main problem was represented by the fixation of the cup, given the retroverted acetabulum along with the elevated rotation centre due to the dysplastic hips. In the case two, a 52 years woman presented dysplastic osteoarthritis of the left hip. A conventional hemispherical cup (Alloclassic-Allofit, Zimmer-Biomet) was placed in the retroverted acetabulum combined with a cementless stem (Fitmore A, Zimmer-Biomet) attached at the metaphyseal proximal femur bone.

**Conclusion:**

Our results suggest that THA may be a good strategy to manage advanced hip osteoarthritis in patients with dysplasia and congenital pubic diastasis.

**Level of evidence:**

IV, case series.

## Background

Bladder exstrophy is rare malformation belonging to the inferior coelosomies [1]. These malformations are characterized by an incomplete closure of the lower abdominal wall during the embryonic development [2]. Bladder exstrophy has a prevalence of approximately one over 30,000 newborns, with higher prevalence in girls [3]. Bladder exstrophy is commonly associated with a wide range of genitourinary, musculoskeletal, and intestinal malformations [4]. Among the musculoskeletal abnormalities, symphysis diastasis, hip dysplasia, and rotational abnormalities (e.g. external rotation of the posterior pelvis and iliac wings, acetabular retroversion) are common [5–8]. Shortening of the pubic bones is also common [9, 10]**.** A prompt closure of the pubic diastasis avoids degeneration of the inner membrane of the bladder [11]. To achieve the closure a direct suture is preferred [4]. Osteotomies are deserved for large diastases: external iliac osteotomy of the sacroiliac joint followed by internal bending of the ileum; osteotomy of the proximal end of the greater sciatic notch followed by internal rotation of the distal bone section; osteotomy of the lateral pubis followed by approximation; dorsal and coronal osteotomy laterally to the sacroiliac joints and distal to the sciatic notch, positioning of bone fragments, and fixation of the pubic bones with a metal plate [4, 12–14].

Little is known about patients who underwent bladder exstrophy repair without pelvic diastasis closure. Retroversion of the dysplastic acetabulum, rotation of the posterior part of the pelvis and iliac wings, and instability of the pelvic ring are the most challenging aspects which the orthopaedic surgeon must be aware when facing these patients [15, 16]. Hence, we presented a case series of two adults who underwent surgical bladder exstrophy repair but with neglected closure of pelvis diastasis. Both patients presented severe coxarthrosis due to dysplasia and underwent total hip arthroplasty (THA).

## Case presentation

### Case one

**Fig. 1:**
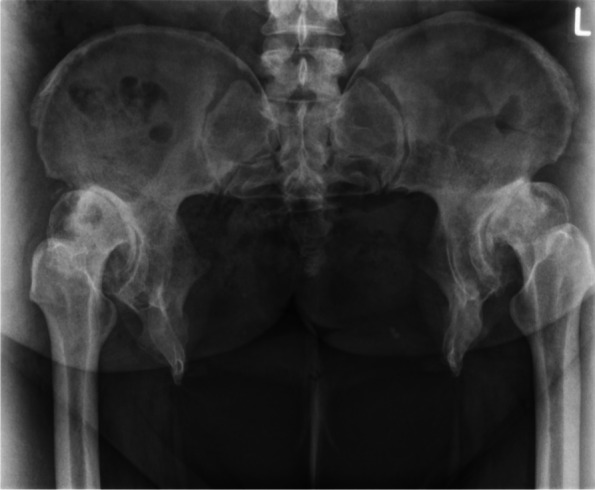
Antero-posterior radiography of the pelvis which shows advanced osteoarthritis secondary to hip dysplasia of both sides combined with a congenital pubic diastasis of 18 cm

A 35 years old woman with a BMI of 46,7 kg/m^2^ was admitted at our department with severe pain in both hips. She underwent multiple surgical interventions to reduce bladder exstrophy. An external urinary kidney catheter was in place. The patient used a wheelchair even for shorter distances. Mobilization waddling gait with crutches shorter than 10 m. The range of motion of both hips were considerably impaired (Right: ext./flex 0°/0°/70°, internal/external rotation in 70° flexion: 0°/0°/10°; abduction adduction 20°/0°/10°. Left: ext./flex 0°/0°/60°, internal/external rotation in 70° flexion: 0°/0°/5°, abduction adduction: 10°/0°/10°). Radiographies (Fig. 1) demonstrated severe bilateral hip osteoarthritis grade III, according to the Tönnis classification [17], grade II femoral head dislocation, according to the Crowe classification [18], 18 cm width pubic diastasis, and shortening of the anterior ischiopubic segment.

**Fig. 2:**
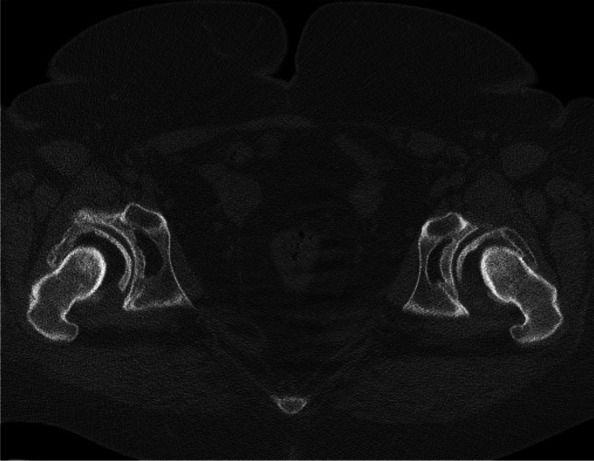
Sequence of the computed tomography of the pelvis which shows the acetabular retroversion combined with external rotation of the posterior pelvis and iliac crests

Computed tomography (CT) (Fig. 2) showed increased external rotation of the posterior pelvis and iliac wings, combined with 12° right and 2° left acetabular retroversion. In 2011 a two-staged THA was performed, with 3 months interval between the two interventions.

**Fig. 3:**
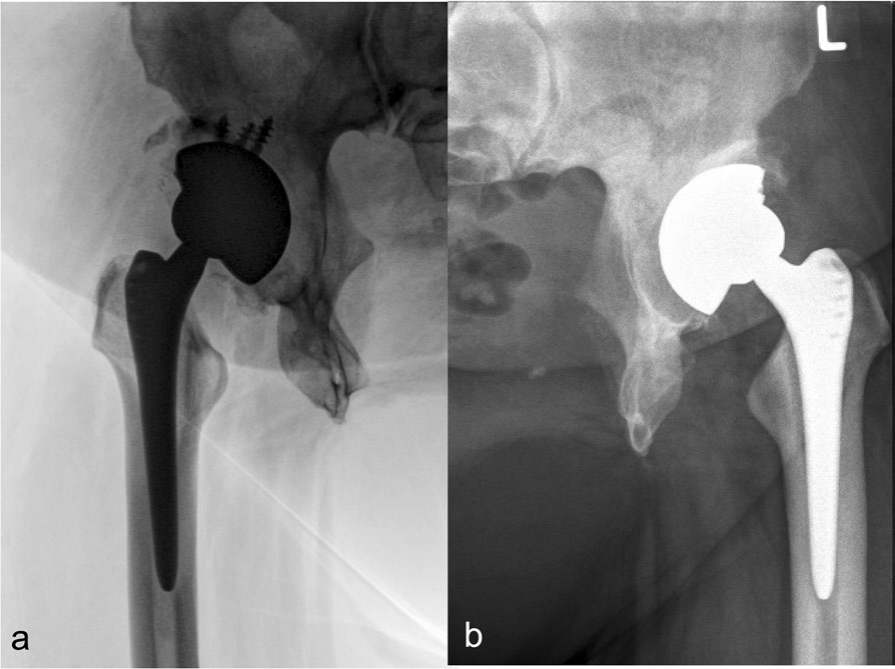
**a**) Postoperative radiography of the right hip with the osteoconductive cup (TM modular cup) size 56 mm with an additional anteverted liner 20° combined with distally fixed stem (Alloclassic-SL); **b**) Postoperative radiography on the left hip at 3 months

The senior author (C.G.) did both operations. A lateral approach with in supine position was used. An osteoconductive cup with high porosity to enhance primary fixation and optimized secondary osteointegration (Trabecular Metal™ modular cup, Zimmer-Biomet GmbH, Winterthur, size 56 mm) was placed with an additional anteverted liner 20° to maintain stability and prevent dislocation (Fig. 3a). Two screws were tightened to increase primary stability of the cementless cup on the right side, while none was required in the left hip (Fig. 3b). On the femoral side a distally-fixed stem Alloclassic-SL (Zimmer-Biomet GmbH, Winterthur) was used to reconstruct anteversion of the proximal femur.

**Fig. 4:**
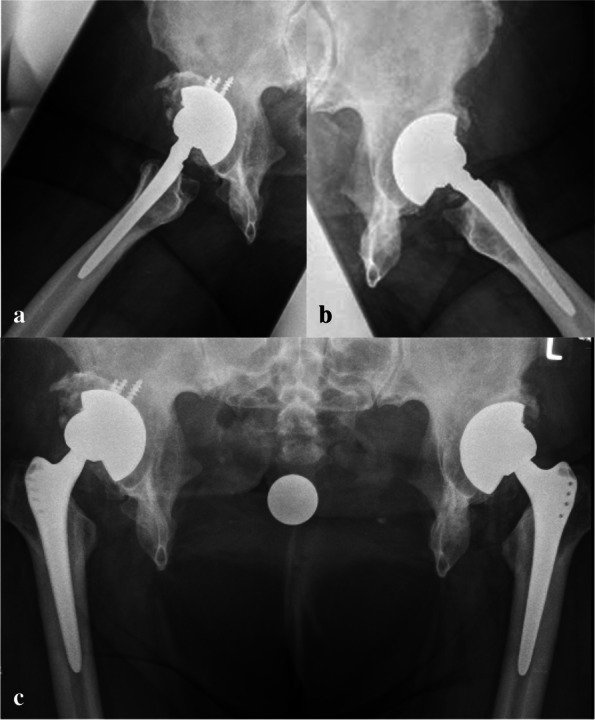
**a**, **b**) 9 years postoperative radiographic control demonstrated no signs of loosening of the acetabular or femoral component; **c**) 9 years postoperative radiographic control in lateral approach evidenced no signs of loosening of the implants

Average surgical duration was 75 min, the intraoperative blood loss was 300 ml. No drains were used. The post-operative rehabilitation started 24 h post-operatively. Full weightbearing was allowed. Passive and active hip motion started at 48 h postoperatively. From the third postoperative day, patients were allowed to stand, and walking re-education program starts with a fully trained physiotherapist. The patient was discarded at sixth postoperative day in a rehabilitation clinic. After 3 months from the last THA, the patient presented had no pain, declaring herself very satisfied with the result. The walk distance with one left crutch increased to 200 m. Similar results were found at last follow-up at 9 years. Radiographic control evidenced no sign of loosening of the components or heterotopic ossification (Fig. 4a, b). The Harris hip Score [19] increased from 14 points preoperatively to 68 at 9 years follow-up. The postoperative right cup inclination and anteversion were 67.3° and 0°, respectively. At last follow-up, both inclination and anteversion remained almost unchanged (67.5° and 0°, respectively). Similarly, also the left cup remained unchanged (inclination 69.1° versus 69.2°; anteversion 0° versus 0°). No difference was found in the width of pubic diastasis post-operatively to last follow-up (18.0 to 18.1 cm). No complication was experienced by the patient.

### Case two

**Fig. 5:**
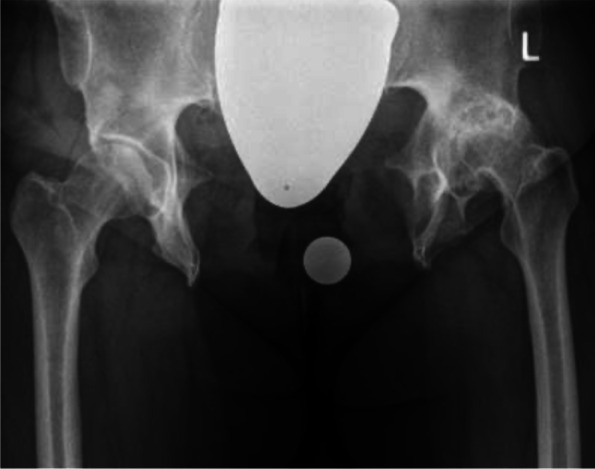
Antero-posterior radiography of the pelvis which shows advanced osteoarthritis secondary to hip dysplasia combined with a congenital pubic diastasis of 14.6 cm

**Fig. 6:**
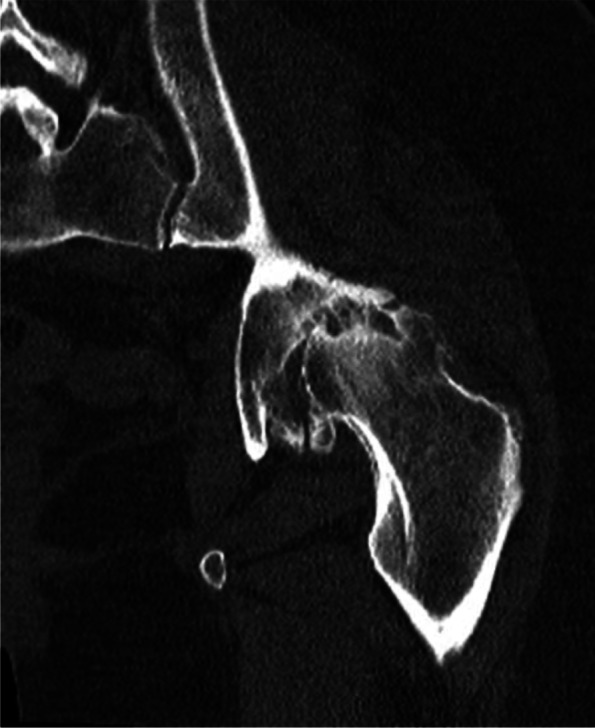
Sequence of the computed tomography of the pelvis which shows the acetabular retroversion combined with external rotation of the posterior pelvis and iliac crests

The second case was a 52 years old woman with prior multiple abdominal operations and a pubic catheter due to congenital bladder aplasia. The patient worked at the time of the surgery as anaesthetist, and declared herself satisfied with work activity and quality of life. The only reason for seeking orthopaedic advice was increased left hip pain and decreased walking distance. The range of motion was considerably impaired (ext./flex: 10°/0°/80°; internal/external rotation in 70° flexion: 10°/0°/15°; abduction adduction: 30°/0°/15°). Radiographically advanced osteoarthritis due to severe dysplasia combined with a congenital pubic diastasis of 14.6 cm (Figs. 5 and 6) was evidenced.

Computed tomography (CT) (Fig.6) showed increased external rotation of the posterior pelvis and iliac wings, combined with 18° right and 3° left acetabular retroversion.

**Fig. 7:**
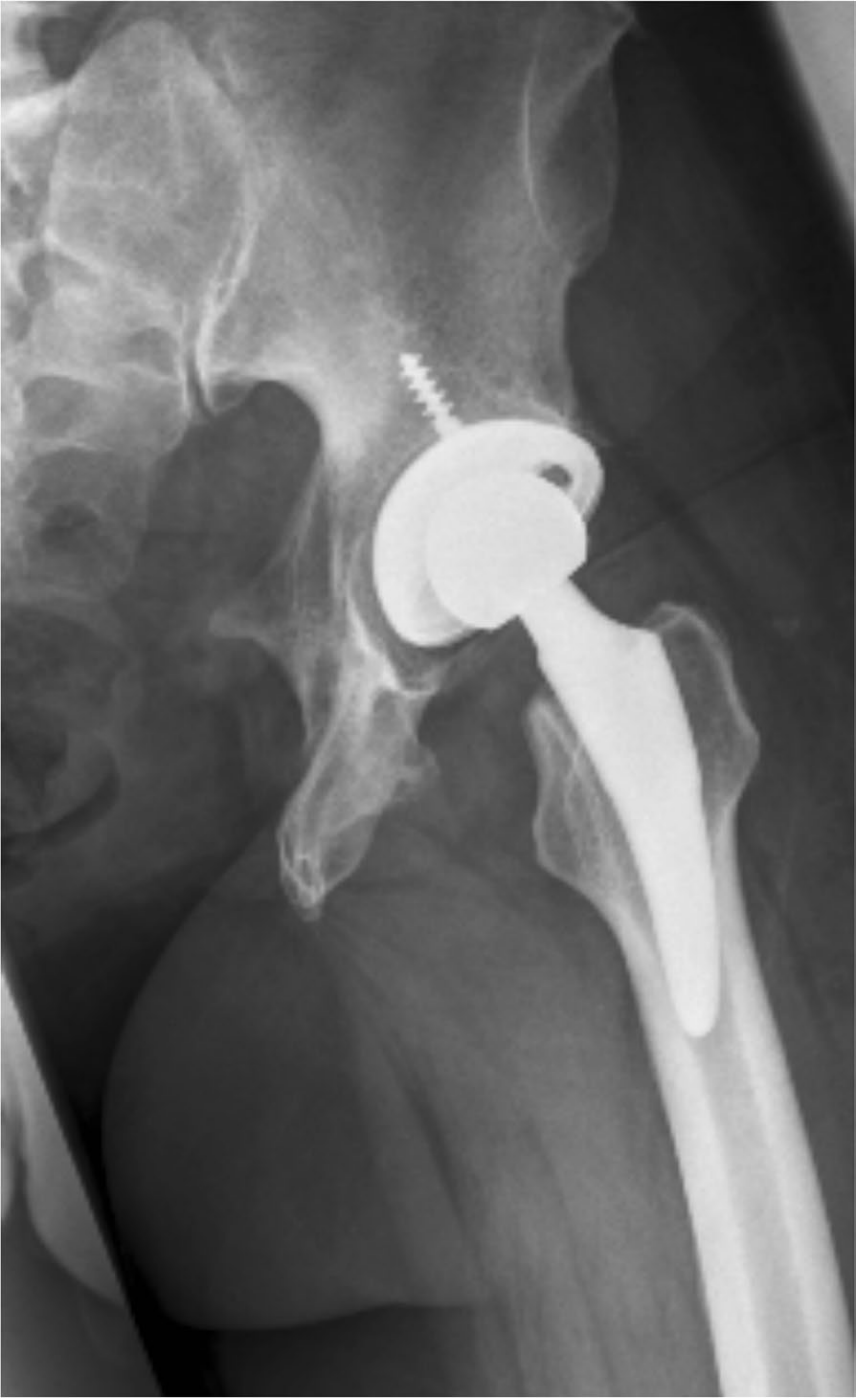
5-days postoperative antero-posterior radiography of the pelvis which shows conventional cup (Allofit) with a metaphyseal fitted stem (Fitmore)

The senior author (C.G.) did the THA. A lateral approach with patient in supine position was used. Average surgical duration was 57 min, the average total intraoperative blood loss was 200 ml. A cementless conventional cup (Allofit, Zimmer-Biomet GmbH, Winterthur) with a metaphyseal fitted stem (Fitmore, Zimmer GmbH, Winterthur) was performed (Fig. 7).

No drains were used. The post-operative rehabilitation started 24 h post-operatively. Full weightbearing was allowed. Passive and active hip motion started at 48 h postoperatively. From the third postoperative day patients were allowed to stand, and walking re-education program starts with a fully trained physiotherapist. The patient was discarded at 8 days after the procedure in a rehabilitation clinic. At 6 months, the patient has no pain in the operated hip, declaring herself satisfied with the operation. Walking distance was unlimited and she could return to work without any impairment. The Harris Hip Score improved from 40 points preoperatively to 80 at last follow-up. At last follow-up, both cup inclination and anteversion remained almost unchanged (+ 1.0° and + 0.5°, respectively). No difference was found in the width of pubic diastasis post-operatively to last follow-up (0.1 cm). No complication was experienced by the patient.

## Discussion and conclusion

Congenital pubic diastasis combined with bladder exstrophy is an embryologic malformation which results in a complex deficit of the anterior midline. The improper development of the inner abdominal wall led to prenatally fascia rupture, causing the skeletal and urogenital malformations. In severe cases, the intestinal system is affected with cloacal malformation, in which the terminal part of the rectum ends in the urethra. Along with pelvis diastasis, many patients present associated rotational anomalies, shifting dorsally the axes of the sacrum and ileum. Furthermore, the posterior rotation of the acetabulum also affects the biomechanics of gait and weights distribution, leading in some cases to early onset osteoarthritis. Currently, various procedures to correct the skeletal malformation were described [12–14]. However, these osteotomies are typically performed during early childhood [8, 20]. In skeletally mature patients ischiopubic osteotomy to correct the diastasis is not possible. This opens concerns regarding the management of these patients, in which diastasis is still present in the adult age. Our results suggested that patients with advanced hip osteoarthritis due to dysplasia combined with congenital pubic diastasis may benefit from THA. Moreover, patients maintain a satisfying outcome over the years, almost comparable with patients without diastasis. Finally, no enlargement of the pubic diastasis was detected, and the position of the cup remained unchanged at last follow-up.

The natural history of this malformation, from an orthopaedic point of view, seems to not affect substantially the quality of life and activities daily living. However, given the limitation of this study, these hypotheses are not fully generalizable. We were able to identify only two studies which described the outcomes of patients with congenital pubic diastasis treated with THA [15, 16]. Both studies reported comparable outcomes, recommending THA as treatment for these patients. However, they also included a limited number of procedures and potentially biased results.

Given the lack of the anterior pubis symphysis, along with the retroverted high-angle and stretched acetabulum, these patients presented high pelvic instability. This instability can affect implant survivorship and increase the risk of failure and dislocations. The severe dysplasia of the retroverted acetabular grove in both cases represents the most challenging problem to overcome surgically. In our cohort of patients, the acetabulum presented enough depth to ensure cup components stabilization. The retroverted acetabulum can be compensated by a neutral position of the acetabular cup with elevated liner to avoid femoral head dislocation, while the anterior rim reduction helps preventing impingement. Greater acetabular retroversion, pelvis extrarotation, and insufficient acetabular depth may stress acetabular component fixation. CT helps to investigate the degree of retroversion of iliac crests, acetabular malorientation, and depth of the acetabular cavity. Moreover, CT helps to predict the position of the acetabular components and to plan the restoration of the hip rotating centre in the acetabular groove [21]. In the first case we used an osteoconductive trabecular metal cups to enhance stability [22]. In the late 90’s first biomechanical testing of osteoconductive trabecular metal cups presented a high biomechanical stability for cancellous and cortical bone structures [23]. Primary stability and secondary osteointegration will be enhanced by osteoconductive trabecular metal cups, reducing the contact with the acetabular bony cavity [24]. An anteverted liner can be placed to reduce the risk of dislocation, allowing to place the cup with a greater inclination. On the femoral side, given its to distal fixation characteristics, a straight rectangle stem was used. The anteversion of the femoral side can be corrected with regard to the position of the stem. The second case had better position of the acetabulum and proximal femur geometry. A conventional cup with screw fixation presented enough stability for secondary osteointegration. Overall, the short and long-term results were successful. The key success might be the reconstruction of hip rotation centre with the acetabular component. In both cases anterior rim trimming was necessary to avoid THA impingement.

This study has certainly limitations. First, the limited study size which jeopardises the ability to identify important variables leading to poor outcomes. However, it should be noted that these patients are uncommon in the clinical practice. In this respect the results are not fully generalisable. Allocation was highly biased, and the lack of randomization and blinding design also affected negatively the reliability of our conclusions. Thus, data from the present study must be interpreted with caution. These results encourage future research to overcome current limitations, establishing in larger scale potential benefits of THA on these patients, giving lights of recommendations and possible pitfalls.

Concluding, our results suggest that THA may represent a strategy to manage advanced hip osteoarthritis in patients with dysplasia and congenital pubic diastasis. These results must be considered within the limitations of the present study, and further researches are required to overcome current limitations, establishing in larger scale potential benefits of THA on these patients, giving lights of recommendations and possible pitfalls.

## Data Availability

All data generated or analysed during this study are included in this published article.

## Abbreviations

THA: Total hip arthroplasty
CT: Computed tomography
TM: Trabecular metal
BMI: Body mass index
